# Allogeneic Expanded Human Peripheral NK Cells Control Prostate Cancer Growth in a Preclinical Mouse Model of Castration-Resistant Prostate Cancer

**DOI:** 10.1155/2022/1786395

**Published:** 2022-04-11

**Authors:** Fangming Wang, Xuejiao Dong, Jing Wang, Feiya Yang, Donghua Liu, Jianlin Ma, Shuai Liu, Dehua Chang, Nianzeng Xing

**Affiliations:** ^1^State Key Laboratory of Molecular Oncology, National Cancer Center, National Clinical Research Center for Cancer, Cancer Hospital, Chinese Academy of Medical Sciences and Peking Union Medical College, 100021 Beijing, China; ^2^Department of Urology, National Cancer Center, National Clinical Research Center for Cancer, Cancer Hospital, Chinese Academy of Medical Sciences and Peking Union Medical College, 100021 Beijing, China; ^3^BOE Regenerative Medicine Technology Co. Ltd., 100015 Beijing, China; ^4^Department of Cell Therapy in Regenerative Medicine, University of Tokyo Hospital, Japan; ^5^Department of Urology, Shanxi Province Cancer Hospital, Shanxi Hospital Affiliated to Cancer Hospital, Chinese Academy of Medical Sciences and Cancer Hospital Affiliated to Shanxi Medical University, China

## Abstract

Adoptive allogeneic natural killer (NK) cell therapy has shown promise in treating castration-resistant prostate cancer (CRPC), which is the terminal stage of prostate cancer (PCa) and incurable. Thus, we employed an efficient manufacturing method for the large-scale *ex vivo* expansion of high-quality NK cells from peripheral blood of healthy donors. In the present study, we evaluated the *in vitro* cytotoxicity of NK cells against human PCa cell lines and *in vivo* antitumor activity in a preclinical mouse model of CRPC. CCK-8 results demonstrated that the NK cells exerted potent cytotoxicity against all PCa cell lines *in vitro*. The NK cells were activated when cocultured with PCa C4-2 cells, evidenced by upregulation of the degranulation marker CD107a and secretion of cytokines (TNF-*α* and IFN-*γ*). In a xenograft mouse model of CRPC, the caliper, CT, and ultrasonography examination results showed that the size of tumors treated with NK cells was significantly smaller than that in the control group. Moreover, ultrasonography examination also indicated that the NK cell treatment evidently reduced the blood supply of the tumors and HE staining results demonstrated that the NK treatment increased the proportion of necrosis in the tumor specimen compared to PBS treatment. Meanwhile, the NK cell treatment did not cause significant serum IL-6 elevation. Therefore, our study suggested that the expanded NK cells exhibited significant cytotoxicity against PCa cell lines *in vitro* and excellent therapeutic efficacy against CRPC in a xenograft mouse model, which was of great value for the clinical treatment of CRPC.

## 1. Introduction

Prostate cancer (PCa) has become the second most common cancer and the fifth leading cause of cancer death among men globally [1]. Although patients with localized PCa can be curatively treated with radical prostatectomy (RP) or radiotherapy, almost half of the subjects will experience recurrence [2]. Consequently, many patients will advance to castration-resistant prostate cancer (CRPC), which remains incurable [3]. In the last decade, immune checkpoint inhibitors (ICIs) have revolutionized the treatment landscape of several malignancies. Unfortunately, recent trials assessing the treatment effect of ICIs on PCa have shown unsatisfactory results. Until now, there is only one anti-PD-1 drug, pembrolizumab, which is approved for the treatment of metastatic CRPC patients with microsatellite instability high (MSI-H) [4]. The disappointing efficacy of ICIs on PCa is likely due to an immunologically cold tumor microenvironment (TME) with minimal T cell infiltration, low expression of programmed death ligand-1 (PD-L1), low expression of neoantigens, and reduced or loss expression of HLA class I [5–7].

Adoptive natural killer (NK) cell therapy is an optional immunotherapy for the treatment of CRPC with low or no HLA class I that are no longer recognized by T cells. Different from CD8^+^ T cells, NK cells express germline-encoded activating and inhibitory receptors to detect altered or missing “self,” rather than express antigen receptors which are generated by somatic gene rearrangement [8]. Thus, NK cells are a crucial complement to T cell-mediated cytotoxicity and appropriate candidates for off-the-shelf adoptive immunotherapy due to no need for HLA matching before targeted killing [6]. Besides, NK cells have the advantage to turn the mentioned “cold TME” into “hot” one as follows: (1) the infused NK cells reach the tumor site and directly lyse cancer cells; (2) NK cells secrete inflammatory cytokines such as interferon-*γ* (IFN-*γ*) and tumor necrosis factor-*α* (TNF-*α*), to recruit CD8^+^ T cells and promote its cytotoxicity; and (3) NK cells recruit dendritic cells (DCs) at tumor sites through the release of cytokines and chemokines [9, 10], which can present tumor antigens and prime CD8^+^ T cells. The conversion of “cold TME” to “hot” ones will pave the way for the efficacy of ICI treatment.

Quantity and quality of donor-derived NK cells are bottlenecks for adoptive NK cell therapy. Thus, *ex vivo* expansion of functional NK cells is the most important step in developing NK cell therapy. Human peripheral blood mononuclear cells (PBMCs) have been used as one of the NK cell sources for clinical applications, and previous studies have used various kinds of NK cell expansion methods to develop NK cell therapy [11]. The feeder cells have been used as an essential method for expanding NK cells. However, the application of feeder cells might cause the problems of infectious diseases and product quality control issues. In this study, we employed a reliable manufacturing system to expand vast quantities of high-quality NK cells with the cytokine combination rather than feeder cell method. The obtained NK cells were shown to have strong cytotoxicity against PCa cell lines and control prostate cancer growth in a preclinical mouse model of CRPC.

## 2. Materials and Methods

### 2.1. Cell Lines and Culture

The human PCa cell lines C4-2/LNCaP/PC-3/DU-145 were purchased from the American Type Culture Collection (ATCC). The mentioned cell lines were cultured in Roswell Park Memorial Institute (RPMI) 1640 medium (Corning, USA) supplemented with 10% (*v*/*v*) fetal bovine serum (FBS) (Corning), 1% penicillin/streptomycin (Solarbio, China), and nonessential amino acid (Corning), at 37°C in a humidified incubator with 5% CO_2_.

### 2.2. Preparation of NK Cells

The study was performed in accordance with the Declaration of Helsinki and was approved by the ethical committee of Cancer Hospital, Chinese Academy of Medical Sciences, and Peking Union Medical College. Blood samples were collected from a peripheral vein of healthy donors after overnight fasting according to a standardized protocol. PBMCs were cultured in OpTmizer™ CTS™ T-cell expansion SFM (Invitrogen, MD) containing 1000 IU/ml human IL-15, 700 IU/ml human IL-2, 0.01 KE/ml OK432 (T&L Biology Technology Co. Ltd.), and 10% Cell-Vive™ Xeno-Free Serum Substitute (BioLegend). T75 cell culture flasks were precoated with anti-human CD16 and anti-human CD3 monoclonal antibodies (BioLegend). After 3–4 days of cultivation for activation, cells were transferred into the antibody-free culture flask in maintaining medium, which was OpTmizer supplemented with IL-2 (Sino Biological) and IL-15 (Sino Biological) as well as 10% Cell-Vive™ Xeno-Free Serum Substitute and cultured for 11 days. Fresh maintaining medium culture medium was added to the flask every 2–3 days according to the cell number, and the cell number was kept at 0.8–1 × 10^6^ cells/ml. All cell culture processes were performed at 37°C in a humidified incubator with 5% CO_2_. The following fluorescently conjugated antibodies were used for the analysis of NK cells: anti-CD56 (BioLegend) and anti-CD3 (BioLegend). Harvested cells were suspended in serum-free freezing media (NutriFreez® D10 Cryopreservation Medium, BI) to make 1 × 10^8^ cells/ml for cell cryopreservation. Frozen NK cells were preserved in liquid nitrogen for 0–12 months. Cells were thawed in a 37°C water bath and slowly diluted with RPMI1640. The NK cell count and viability were assessed by acridine orange/propidium iodide (AO/PI) staining using an automatic fluorescence cell counter (Countstar).

### 2.3. Cytotoxicity Examination of NK Cells

The C4-2/LNCaP/PC-3/DU-145 cells in the logarithmic growth phase were added to a 96-well plate with 10000 cells/well, respectively. On the second day, NK cells were added as an effector with the target ratio of 10 : 1/5 : 1/2 : 1/1 : 1/0.5 : 1/0.2 : 1/0.1 : 1/0.05 : 1 and tested in quadruplicate. Mixed incubation was done for 24 h. Then, the supernatant was removed out of the plate and all adherent cells were washed twice by 100 *μ*l PBS. Then, CCK-8 (TRANS, Code#FC101-03) was added to complete medium, followed by incubation for another 3 h at 37°C. The blank controls were set with only CCK-8 and complete medium. Microplate reader measured the absorbance of the wells at 450 nm wavelength. The killing rate was calculated as follows: killing rate = (OD_experiment_ − OD_blank_/OD_negative_ − OD_blank_) × 100%.

### 2.4. CD107a Expression

The expanded NK cells were incubated with or without C4-2 cells at a 1 : 1 ratio for 20 hours at 37°C. NK cells were collected and treated with a GolgiStop protein transport inhibitor (BD Bioscience) for 2 hours, followed by a single wash with Cyto-Fast Perm/wash 1x solution (BioLegend). Next, the cells were resuspended and Cyto-Fast Fix/Perm solution (BioLegend) was added and incubated for 20 min in the dark at room temperature. After being washed once, cells were immunostained with anti-CD107a (LAMP-1) (BioLegend) for 10 min. After being washed twice, the cells were resuspended in 200 *μ*l PBS and the expression of CD107a was analyzed by flow cytometry (Merck, easyCyte HT).

### 2.5. Cytokine Release Assay

INF-*γ* production and TNF-*α* production were measured by human ELISA INF-*γ* and TNF-*α* kits (BioLegend) following the manufacturer's instructions. Briefly, different numbers of NK cells were seeded in triplicate together with 1 × 10^4^ C4-2 cells in a 96-well bottom plate. Cytokine secretion was measured after 12 h of incubation. Negative and positive controls were represented by NK cells that remained unstimulated or were treated with 10 *μ*g/ml of PHA-M (Sigma-Aldrich). The concentrations of interleukin- (IL-) 6 in serum collected from mouse tail veins were assessed using a human IL-6 ELISA Kit (Invitrogen) following the manufacturer's protocol.

### 2.6. Flow Cytometry

Single-cell suspensions were prepared. All human PBMC or PBMC-derived immunocytes were blocked with human anti-CD16 first. Single cells then were stained with a combination of following antibodies purchased from BioLegend: anti-CD3 (OT3), anti-CD56 (NCAN), anti-NKG2D (1D11), anti-MICA/B (6D4), and anti-CD107a (H4A3). Data were acquired by a flow cytometer (LSRFortessa, BD Biosciences). The results were analyzed with FlowJo software (version 10) (Treestar, USA).

### 2.7. Mice and Procedures

**Figure 1:**
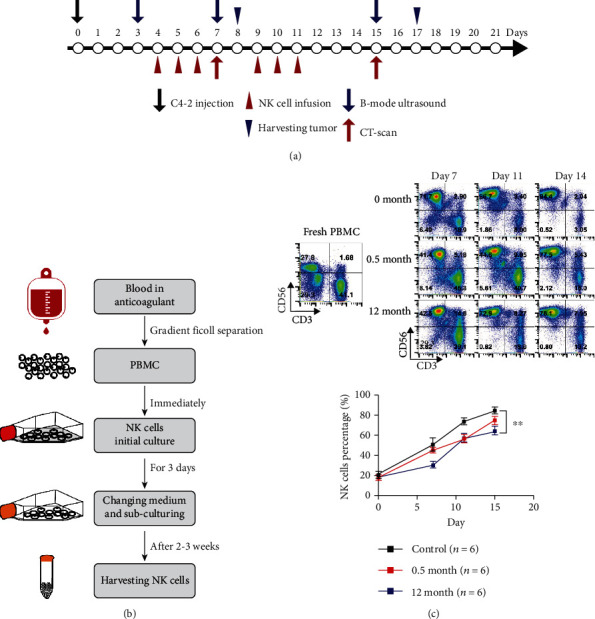
The *in vivo* experiment arrangements and NK cell production with validation. (a) Time schedule for *in vivo* experiment. The mice were subcutaneously injected with C4-2 cells on day 0 and randomly grouped into treatment and control groups (*n* = 12 in each group). Treatment started on day 4 and contained 2 cycles: days 4–6 for the first cycle and days 9–11 for the second cycle. The ultrasonography was performed on days 3, 7, and 15, and Micro-CT scan was performed on days 7 and 15 to evaluate the tumor size change. On day 8, six mice in each group were randomly chosen and killed after the first cycle of treatment. The remaining six living mice in each group continued to accept the second cycle of treatment and were sacrificed on day 17. (b) Overview of the steps leading from human blood to peripheral blood NK cells. NK cells efficiently differentiate and expand in culture and could be harvested in 2–3 weeks. (c) Representative flow cytometry plots and summary data (*n* = 3) of NK and T cell percentages. NK cells (CD3^−^CD56^+^) and T cells (CD3^+^CD56^−^) account for 27.8% and 41.1% in fresh PBMC, respectively; on days 7, 11, and 14 after culture, the NK cells (CD3^−^CD56^+^) account for 71.7%, 86.7%, and 94.4% in expanded fresh cells, respectively. The 0.5 month cryopreserved NK cells after days 7, 11, and 14 culture account for 41.4%, 44.6%, and 77.5%, respectively, and the 12 month cryopreserved NK cells after days 7, 11, and 14 culture account for 42.5%, 72.9%, and 78.1% after being thawed, respectively. There is significant difference between the fresh NK cells and thawed 12 month cryopreserved NK cells in NK cell percentages (*n* = 6 independent expansions from 6 healthy donors). Values are expressed as the means ± SD, ^∗∗^*p* < 0.01. PBMC: peripheral blood mononuclear cell.

Five-week-old male NOD/SCID mice were purchased from Beijing Vital River Laboratory Animal Technology Company and housed in a pathogen-free animal facility of the State Key Laboratory of Molecular Oncology of the National Cancer Center. All experimental procedures were performed under guidelines approved by the ethics committee of our hospital and the National Institutes of Health Guide for the Care and Use of Laboratory Animals. For *in vivo* experiments testing NK cell function against CRPC, the mice were subcutaneously injected with 2 × 10^6^ C4-2 cells/mouse on day 0. As shown in Figure 1(a), treatment started on day 4 after mice were randomly distributed into control and NK groups with similar tumor volumes (*n* = 12). Treatment was designed to contain 2 cycles in a 3-day period. 1 × 10^7^ NK cells suspended in 200 *μ*l PBS were injected via the tail vein twice a day, while tumor-bearing mice in the control group received PBS tail vein injection of the same volume on days 4, 5, and 6 for the first (1st) cycle and days 9, 10, and 11 for the second (2nd) cycle. Tumor volumes were evaluated using a caliper 3 times a week according to the standard formula: *L* × *W*^2^/2, where *L* and *W* represent the longest and shortest diameters, respectively. Tumor sizes before and after treatment were also evaluated by ultrasonography (S-Sharp Prospect, Taiwan) and Micro-CT (Quantum FX, Rigaku, Japan) on days 3, 7, and 15 after anaesthesia. Besides, the colored doppler flow (CDF) mode in ultrasonography was used to detect the maximal blood flow velocity (BFV_max_) of the subcutaneous implanted tumor. On day 8, six mice in each group were randomly chosen and sacrificed after the 1st cycle of treatment. The remaining six living mice in each group continued to accept the 2nd cycle of treatment and were killed on day 17. The implanted tumors were harvested, photographed, weighed, and collected for histological examination after the mice were killed. Besides, we collected blood from the tail vein to measure IL-6 levels on day 12 after the 2nd cycle of treatment.

### 2.8. Histology and Quantification of Necrosis

The tumor tissues were fixed with 10% neutral formalin, embedded in paraffin, and then sliced into 4 *μ*m thick paraffin sections. Dewaxed sections were stained with hematoxylin and eosin (HE). Without an integrated cell structure, the necrotic area was stained red under a light microscope. NDP.view2 Viewing software (Hamamatsu Photonics, Shizuoka, Japan) was used to quantify the red necrotic area percentage. These analyses were all conducted in a blinded manner by two experienced pathologists.

### 2.9. Statistical Analysis

The data were expressed as a number (percentage) for categorical variables and means ± SD for continuous variables. The categorical variables were analyzed by *χ*^2^ test or Fisher's exact test where appropriate. The differences between continuous variables were analyzed by the Mann–Whitney *U* test or unpaired *t*-tests. A *p* value < 0.05 (two sided) was considered significant for all tests. The statistical analyses were performed with SPSS version 22.0 software (SPSS Inc., Chicago, IL, USA).

## 3. Results

### 3.1. Characterization of PBMCs and NK Cells in Healthy Donors

In the present study, we established a method for large-scale expansion of functionable NK cells from peripheral blood in 2 weeks. We evaluated the characterization of fresh NK cells derived from PBMCs after 7-, 11-, and 14-day cultures and the corresponding cryopreserved NK cells. The NK cells were efficiently expanded according to our designed protocol (Figure 1(b)). First, we isolated PBMCs from peripheral blood of healthy donors. Six healthy adult donors were enrolled in the study. Three donors were females and three donors were males; the range was 28–45 years. As shown in Figure 1(c), NK cells (CD3^−^CD56^+^) and T cells (CD3^+^CD56^−^) account for 27.8% and 41.1% in fresh PBMC, respectively. From 7- to 14-day cultures, NK cell percentages gradually increased. On day 14, PBMCs stimulated with cytokines were composed of highly enriched CD3^−^CD56^+^ NK cells (85.4 ± 7.7%) with little contamination by CD3^+^ T cells (14.6 ± 1.2%). Moreover, the components of 0.5- and 12-month cryopreserved 14-day cultured NK cells after thawing were relatively stable, with NK cell percentages of 75.5 ± 10.5% and 64.3 ± 9.9%.

### 3.2. Cytotoxicity of NK Cells against PCa Cell Lines *In Vitro*

**Figure 2:**
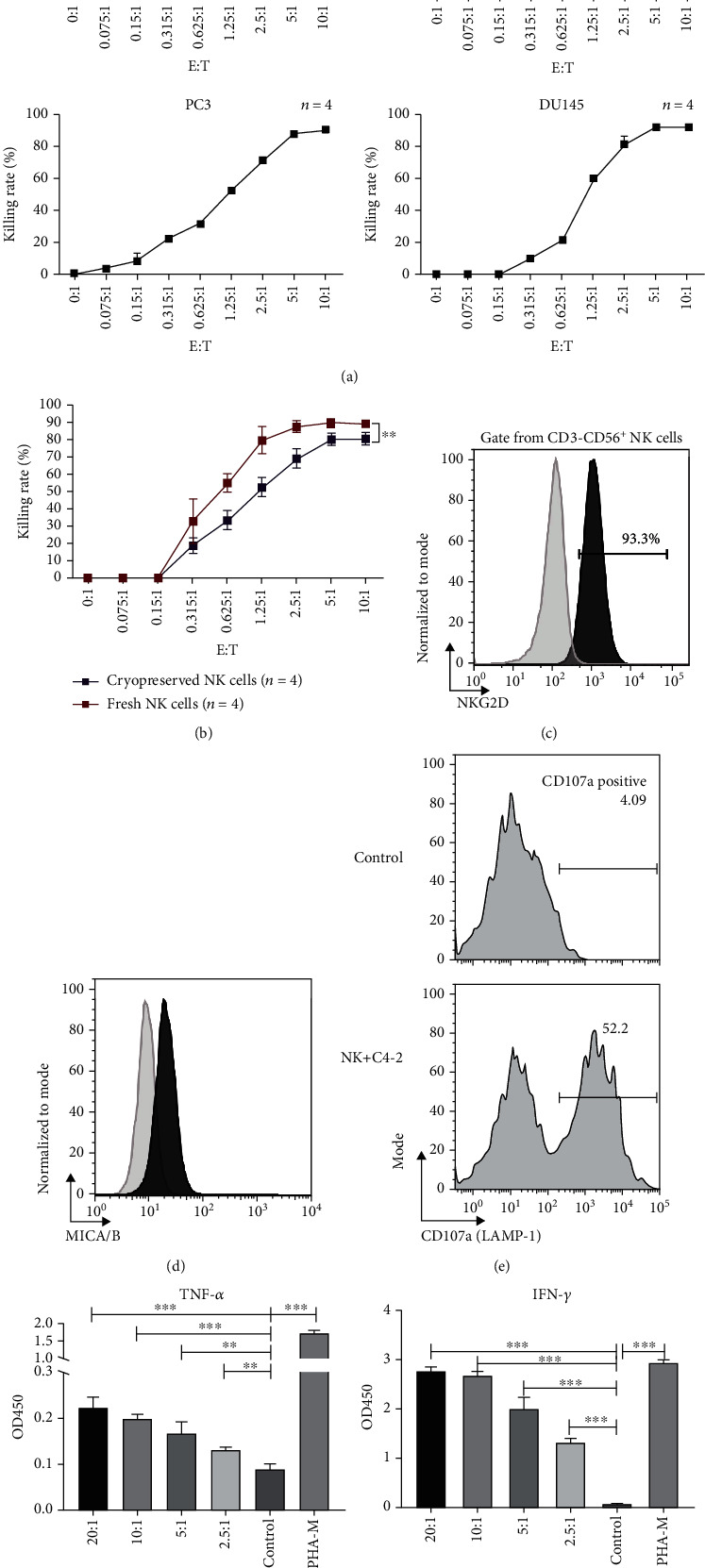
Cytotoxicity, degranulation activity, and cytokine secretion of the expanded NK cells. (a) Killing rates of the NK cells against multiple prostate cancer cell lines including C4-2, LNcap, PC-3, and DU-145 at different E/T ratios after 24 h coculture. Data are shown as means ± SD (*n* = 4 technical replicates). *E*/*T*: effector-to-target ratio. (b) Comparison of killing rates against the C4-2 cell line between fresh NK cells and 1 month cryopreserved NK cells at different *E*/*T* ratios. Data are shown as means ± SD (*n* = 4 technical replicates). ^∗^*p* < 0.05. *E*/*T*: effector-to-target ratio. (c) Representative flow cytometry plots and data of NKG2D expression on CD3^−^CD56^+^ NK cells. NKG2D: NK group 2 member D. (d) Representative flow cytometry plots and data of MICA/B expression on C4-2 cells. MICA/B: MHC class I chain-related protein A/B. (e) Representative flow cytometry plots and data of CD107a expression on the NK cells after being cocultured with C4-2 cells and NK cell alone. (f) IFN-*γ* and TNF levels of the supernatant after the NK cells were cocultured with C4-2 cells at *E*/*T* ratios of 2.5 : 1, 5 : 1, 10 : 1, and 20 : 1 or stimulated with PHA-M for 12 hours. Values are expressed as the means ± SD, ^∗∗^*p* < 0.01; ^∗∗∗^*p* < 0.001. OD: optical density; *E*/*T*: effector-to-target ratio; PHA-M: phytohemagglutinin-M.

The expanded NK cells were assessed for cytotoxicity and cytokine secretion when cocultured with C4-2 cells. The C4-2 cell line had acquired castration-resistant characteristics under the condition of androgen deprivation and represented the CRPC cells. NK cells were cocultured with C4-2 cells at different effector-to-target ratios (*E*/*T*) for 24 hours. CCK-8 results showed that the NK cells exerted potent cytotoxicity against C4-2 cells, the significant cytotoxic effects could be observed at *E*/*T* of 2.5 : 1 and reached a peak at 5 : 1. The cell lysis ratio reached 90.3 ± 0.7% when the *E*/*T* was 5 : 1 (Figure 2(a)). Besides, our data demonstrated that the NK cells exerted strong cytotoxicity against other PCa cell lines including LNCap, PC-3, and DU-145 as well, particularly at high *E* : *T* ratios of 5 : 1 (Figure 2(a)). Moreover, although the direct cytotoxicity against C4-2 cells of cryopreserved NK cells was lower than that of the fresh counterparts (killing rate 80.4 ± 3.7% vs. 90.3 ± 0.6% when *E*/*T* = 5 : 1, *p* = 0.002), the thawed cryopreserved NK cells still showed robust cytotoxicity (Figure 2(b)). Then, we measured NK group 2 member D (NKG2D) on the NK cells and MICA/MICB (ligand of NKG2D) on C4-2 cells and observed that 93.3% of the NK cells expressed NKG2D (Figure 2(c)) and 28.1% of the C4-2 cells expressed MICA/MICB (Figure 2(d)). In addition, we observed that the expression of CD107a, a marker of NK cell degranulation, increased significantly on NK cells cocultured with C4-2 cells, compared to NK cells alone, indicating the vigorous degranulation activity of NK cells (Figure 2(e)). Next, IFN-*γ* and TNF-*α* levels of the supernatant were measured when the NK cells were cocultured with C4-2 cells for 24 hours at *E*/*T* ratios of 2.5 : 1, 5 : 1, 10 : 1, and 20 : 1. The results demonstrated that the NK cells released more TNF-*α* and IFN-*γ* than the negative control when cocultured with C4-2 cells; besides, the cytokine secretions were positively correlated with the *E*/*T* ratio (Figure 2(f)).

### 3.3. The Antitumor Activity of NK Cells on C4-2 Cell Xenografts *In Vivo*

**Figure 3:**
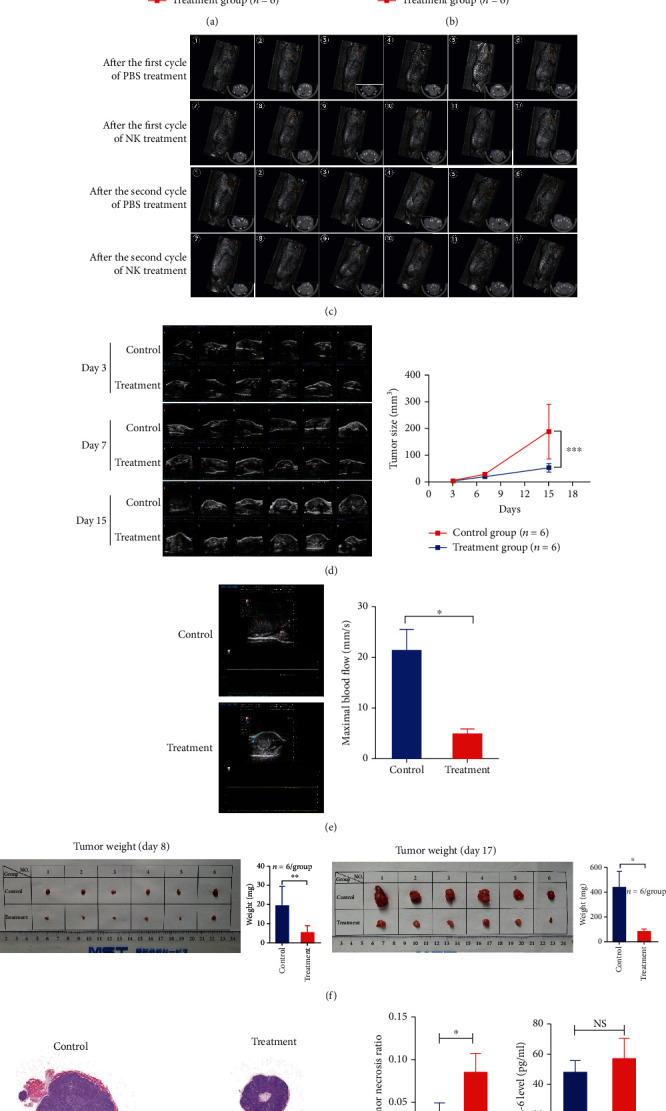
The measurement data of implanted tumors and IL-6 levels in the NK cell treatment and control groups *in vivo*. (a) Tumor size measured by a caliper in the treatment and control groups over the whole course of treatment (*n* = 6 for each group). (b) Body weight in the treatment and control groups over the whole course of treatment (*n* = 6 for each group). (c) The CT scan of each mouse after the first and the second cycles of treatment in NK cells and control groups; the tumors were labelled with orange circles in three-dimensional reconstruction image and with blue circles in the cross-section. (d) Tumor size measured by ultrasonography examination in the treatment and control groups before treatment (day 3), after the first (day 7) and the second cycles (day 15) of treatment (*n* = 6 for each group). (e) The maximal blood flow measured by colored doppler-flow mode of ultrasonography in the treatment and control groups on day 15. (f) Images and weight data of harvested tumors from the mice in the treatment and control groups on days 8 and 17 (*n* = 6 for each group). (g) HE examination and necrosis area percentages of tumor specimen in the treatment and control groups on day 17 (*n* = 6 for each group). (h) Serum IL-6 levels in the treatment and control groups on day 12. Statistical significance was determined by unpaired *t*-test. Values are expressed as the means ± SD, ^∗^*p* < 0.05, ^∗∗^*p* < 0.01, and ^∗∗∗^*p* < 0.001; NS: not significant; IL-6: interleukin-6.

To begin with, the subcutaneously implanted C4-2 cells successfully formed visible tumors in the NOD/SCID mice, which lack B, T, and NK cells on day 3 after inoculation. Our data demonstrated that NK cell treatment significantly reduced the tumor size measured by a caliper compared with the control group (Figure 3(a)), while the mouse body weight of the two groups showed no significant difference (Figure 3(b)). In consistency with the abovementioned findings, the CT scan results of each mouse showed that the tumor volumes began to show a significant reduction after the 1st cycle treatment of NK cells and the 2nd cycle treatment of NK cells augmented the volume difference between the two groups (Figure 3(c)). Moreover, the ultrasonography results showed that there was no difference between the two groups on day 3 before treatment commencement and the tumor size of the treatment group was significantly smaller than that of the control group on days 7 and 15 (Figure 3(d)). The CDF examination showed that the BFV_max_ of the treatment group was significantly lower than that of the control group (Figure 3(e)), indicating that NK cells reduced the blood supply of the implanted tumor. After harvesting the tumors from the mice on days 8 and 17, we weighed the tumors and found that the masses of tumors in the treatment group were evidently lower than those in the control group (Figure 3(f)). HE examination demonstrated that the necrosis area percentages of the tumor specimen in the treatment group were significantly higher than those in the control group (Figure 3(g)). Besides, serum IL-6 of mice in the NK cell treatment group was slightly higher than that in the control group but showed no significant difference (57.1 ± 5.2 vs. 48.9 ± 2.6, *p* = 0.189) (Figure 3(h)).

## 4. Discussion

Our study employed a clinical culture system that would produce large numbers of functional NK cells from human peripheral blood for adoptive immunotherapy and firstly demonstrated that allogeneic peripheral NK cells exhibited significant direct cytotoxicity to several PCa cell lines *in vitro* and strong antitumor activity in a xenograft mouse model of CRPC *in vivo* with safety. The expanded NK cells could directly lyse PCa cells and secrete important inflammatory cytokines including IFN-*γ* and TNF-*α*. Therefore, the present study implicated that the allogeneic NK cells derived from peripheral blood had great value in clinical application for CRPC patients.

As present, androgen deprivation therapy, radiotherapy, RP surgery, and chemotherapy are available for treating PCa. Localized PCa can be treated curatively by surgery or radiation, but once PCa spreads outside the prostate, cancer cells could be hardly removed completely by the mentioned therapies under the circumstance of immune system dysfunction. Adoptive NK cell therapy has been known to boost host immunity against cancer and has shown promise in treating cancer because NK cells can fight against malignant cells without prior exposure to antigens and with low risk for causing cytokine release syndrome (CRS) or graft versus host disease, compared with T cells [12]. Autologous NK cell infusions were firstly considered applied in clinical practice because they could be conveniently obtained from the peripheral blood of the patient's own; however, studies of this method have revealed that the infused NK cells did not exhibit antitumor effects against hematological or solid tumors, perhaps due to the lack of mismatching between inhibitory receptors of the NK cells and autologous tumor cells [13, 14]. The findings prompted a conversion from autologous to allogeneic NK cell therapy. There are different sources of allogeneic NK cells, such as NK-92 cell lines, peripheral blood-derived NK cells, iPSC- (induced pluripotent stem cell-) derived NK cells, umbilical cord blood NK cells, chimeric antigen receptor- (CAR-) engineered NK cells, haploidentical NK cells, and adaptive NK cells, which have their own pros and cons. Generally speaking, there are two objectives of developing allogeneic NK cell therapy: choosing a suitable source that could provide NK cells with enough purity and quantity and taking measures including improvement of the culture formula to potentiate NK cell cytotoxicity [12]. There were phase I clinical trials using the NK-92 cell line to treat refractory or relapsed hematological malignancies in an allogeneic setting, showing safety and evidence of efficacy of this adoptive NK-92 cell therapy [15, 16]. However, there is a lack of preclinical or clinical studies that apply allogeneic NK cells for the treatment of PCa. Montagner et al. [17] reported CAR NK-92 cell therapy against PCa in mouse tumor models based on the CAR construction specifically recognizing the human prostate-specific membrane antigen, which is overexpressed in PCa cells. However, tumorigenicity of NK-92 limited its application in clinical use although it was reported to be safely injected after irradiation. In our study, we employed a large-scale *ex vivo* NK cell expansion method, with which we can isolate 2 × 10^6^ PBMCs from one milliliter blood and performed at least 100-fold expansion of NK cells in two weeks. For example, assuming that one healthy person donated 200–400 ml blood, we could ultimately obtain 20–40 billion functional NK cells after expansion in two weeks (2 × 10^6^/ml × 200–400 ml × 100 = 2–4 × 10^10^). In a recent clinical trial evaluating the efficacy of allogeneic NK cells in treating patients with non-small cell lung cancer, the enrolled patients continuously received 1–3 courses of allogeneic NK cell treatment and one course of NK cell therapy included 24 billion NK cells (12 billion/cycle × 2 cycle) [18]. Besides, we searched for NK dosages in clinical trials involving NK cell treatment for solid tumors [19] and confirmed that our method could provide enough expanded NK cells that can meet the clinical requirements for treating patients with PCa. As for NK cell purity, our data showed that approximately 85.4% of PBMCs were NK cells on day 14 after culture. Moreover, 12 months of cryopreservation only slightly decreases the purity and cytotoxicity of expanded NK cells. There was one study reporting that the peripheral blood NK cell number in patients with relapsed multiple myeloma who received freshly expanded NK cells was higher than in those who received cryopreserved NK cells [20]. Our *ex vivo* NK cell expansion/frozen data were consistent with the report, and the decreased cytotoxicity of cryopreserved NK cells can be compensated with increased dosage. Our method uses cytokines to stimulate PBMCs and further expand NK cells, and the expanded NK cells were evaluated for cytotoxicity and cytokine secretion when cocultured with PCa cell lines. The expanded NK cells exhibited strong cytotoxicity against diverse human PCa cell lines including C4-2, LNCap, PC-3, and DU-145 cells, particularly at higher E/T ratios (≥5 : 1), through vigorous degranulation activity, which was reflected by upregulations of CD107a expression. The release of effector molecules, such as granzyme B and perforin, will directly lyse the target cells. Human PCa cell lines used in our study can represent different PCa stages and have their respective genomic and transcriptional characteristics [21]. Therefore, we inferred that our expanded NK cells would exert strong cytolytic activity against PCa cells at the tumor site when applied to patients with different stages of PCa. Besides, the clinical trial of using those NK cells for the treatment of CRPC is currently in preparation in our center. In addition, the expanded NK cells secreted amounts of IFN-*γ* and TNF-*α* when cocultured with C4-2 cells and those cytokines would play important roles in reshaping the immune profile of the TME. As for the mechanisms of NK cell activation, studies have demonstrated that activating signals mediated by the NKG2D/NKG2DL pathway can override the signals induced by the inhibitory receptors, thereby allowing NKG2D to act as a “master switch” for activating NK cells [22]. NKG2D could recognize MICA/B and the other 6 different NKG2D ligands [23], and the NKG2D signaling pathway can mediate a direct killing effect in NK cells [24]. Our data showed that the NK cells expressed abundant NKG2D and C4-2 cells expressed MICA/B, which partly explained the activation mechanism.

To simulate the NK cell regimen for the treatment of CRPC, we applied 2 cycles of freshly expanded NK cells to mice bearing subcutaneous C4-2 tumors *in vivo*. Tumor burden evaluated by a caliper, CT scan, and ultrasonography showed that both the 1st and 2nd cycles of the expanded NK cells can efficiently control prostate cancer growth. Pathological results demonstrated that 2-cycle treatment of NK cells caused evident necrosis in the harvested tumor specimen, which was further supported by the CDF examination results showing reduced tumor blood supply after NK cell treatment. Adoptive cell therapy can be accompanied by severe side effects such as CRS, in which IL-6 and other cytokines were massively released and caused multiple organ damage [25, 26]. In our study, although the IL-6 level of mouse blood was marginally elevated after NK cell treatment, there was no significant difference between the treatment and control groups. Besides, it was reported that some patients accepting NK-92 therapy developed anti-HLA antibody responses [15, 27], which occurred typically after the second cycle of NK cells and may be related to repeated cycles of high cell doses. However, there was no serious side effects observed in patients with HLA antibodies. Therefore, we inferred that our expanded peripheral NK cells would not cause serious adverse effect due to possible anti-HLA antibody response. Taken together, our data indicated that the allogeneic proliferating human peripheral NK cells have strong antitumor effects against PCa cells, which is reflected by degranulating activity and cytokine release; meanwhile, the NK cells have satisfactory safety when used for PCa treatment.

## 5. Conclusions

In conclusion, we successfully established a method to expand the quantities of human peripheral NK cells *ex vivo* for adoptive immunotherapy; the expanded NK cells even after cryopreservation showed efficient antitumor effects against PCa cells. The expanded NK cells meet the clinical quality standards and can be taken for scheduled “off-the-shelf” use and are currently being applied for a clinical trial of CRPC patients in China. The works presented here has two implications: first, NK cell infusion could restore responsiveness to ICI as they could convert a cold TME into a hot one; thus, the combination of NK cells and ICIs for the treatment of CRPC needs further research; second, the expanded NK cells provide a solid foundation for the construction and production of CAR-engineered NK cells, which could target the tumor antigen specifically.

## Data Availability

Data supporting the findings are available from the corresponding author upon request.

## Abbreviations

PCa: Prostate cancer
CRPC: Castration-resistant prostate cancer
E/T: Effector-to-target ratio
TNF: Tumor necrosis factor
IFN-*γ*: Interferon-*γ*
IL-6: Interleukin-6
BFV_max_: Maximal blood flow velocity
HE: Hematoxylin and eosin
ICI: Immune checkpoint inhibitor
TME: Tumor microenvironment
PD-L1: Programmed cell death-ligand 1
PBMC: Peripheral blood mononuclear cell
DC: Dendritic cell
CCK-8: Cell counting kit-8
NKG2D: NK group 2, member D
MICA/B: MHC class I chain-related proteins A and B
CDF: Colored doppler flow
CRS: Cytokine release syndrome

## References

[1] Sung H., Ferlay J., Siegel R. L. (2021). Global cancer statistics 2020: GLOBOCAN estimates of incidence and mortality worldwide for 36 cancers in 185 countries. *CA: A Cancer Journal for Clinicians*.

[2] Cooperberg M. R., Carroll P. R. (2015). Trends in management for patients with localized prostate cancer, 1990–2013. *Journal of the American Medical Association*.

[3] Paller C. J., Antonarakis E. S. (2013). Management of biochemically recurrent prostate cancer after local therapy: evolving standards of care and new directions. *Clinical Advances in Hematology & Oncology*.

[4] Marcus L., Lemery S., Keegan P., Pazdur R. (2019). FDA approval summary: pembrolizumab for the treatment of microsatellite instability-high solid tumors. *Clinical Cancer Research*.

[5] Bilusic M., Madan R. A., Gulley J. L. (2017). Immunotherapy of prostate cancer: facts and hopes. *Clinical Cancer Research*.

[6] Cichocki F., Bjordahl R., Gaidarova S. (2020). iPSC-derived NK cells maintain high cytotoxicity and enhance in vivo tumor control in concert with T cells and anti-PD-1 therapy. *Science Translational Medicine*.

[7] Carretero F. J., Del Campo A. B., Flores-Martín J. F. (2016). Frequent HLA class I alterations in human prostate cancer: molecular mechanisms and clinical relevance. *Cancer Immunology, Immunotherapy*.

[8] Lanier L. L. (1998). NK cell receptors. *Annual Review of Immunology*.

[9] Barry K. C., Hsu J., Broz M. L. (2018). A natural killer-dendritic cell axis defines checkpoint therapy-responsive tumor microenvironments. *Nature Medicine*.

[10] Böttcher J. P., Bonavita E., Chakravarty P. (2018). NK cells stimulate recruitment of cDC1 into the tumor microenvironment promoting cancer immune control. *Cell*.

[11] Koepsell S. A., Miller J. S., McKenna D. H. (2013). Natural killer cells: a review of manufacturing and clinical utility. *Transfusion*.

[12] Myers J. A., Miller J. S. (2021). Exploring the NK cell platform for cancer immunotherapy. *Nature Reviews. Clinical Oncology*.

[13] Veluchamy J. P., Kok N., van der Vliet H. J., Verheul H. M. W., de Gruijl T. D., Spanholtz J. (2017). The rise of allogeneic natural killer cells as a platform for cancer immunotherapy: recent innovations and future developments. *Frontiers in Immunology*.

[14] Parkhurst M. R., Riley J. P., Dudley M. E., Rosenberg S. A. (2011). Adoptive transfer of autologous natural killer cells leads to high levels of circulating natural killer cells but does not mediate tumor regression. *Clinical Cancer Research*.

[15] Williams B. A., Law A. D., Routy B. (2017). A phase I trial of NK-92 cells for refractory hematological malignancies relapsing after autologous hematopoietic cell transplantation shows safety and evidence of efficacy. *Oncotarget*.

[16] Boyiadzis M., Agha M., Redner R. L. (2017). Phase 1 clinical trial of adoptive immunotherapy using “off-the-shelf” activated natural killer cells in patients with refractory and relapsed acute myeloid leukemia. *Cytotherapy*.

[17] Montagner I. M., Penna A., Fracasso G. (2020). Anti-PSMA CAR-engineered NK-92 cells: an off-the-shelf cell therapy for prostate cancer. *Cell*.

[18] Lin M., Luo H., Liang S. (2020). Pembrolizumab plus allogeneic NK cells in advanced non-small cell lung cancer patients. *The Journal of Clinical Investigation*.

[19] Liu S., Galat V., Galat Y., Lee Y. K., Wainwright D., Wu J. (2021). NK cell-based cancer immunotherapy: from basic biology to clinical development. *Journal of Hematology & Oncology*.

[20] Szmania S., Lapteva N., Garg T. (2015). Ex vivo-expanded natural killer cells demonstrate robust proliferation in vivo in high-risk relapsed multiple myeloma patients. *Journal of Immunotherapy*.

[21] van Bokhoven A., Varella-Garcia M., Korch C. (2003). Molecular characterization of human prostate carcinoma cell lines. *The Prostate*.

[22] Watzl C. (2003). The NKG2D receptor and its ligands-recognition beyond the "missing self"?. *Microbes and Infection*.

[23] Fuertes M. B., Domaica C. I., Zwirner N. W. (2021). Leveraging NKG2D ligands in immuno-oncology. *Frontiers in Immunology*.

[24] Liu H., Wang S., Xin J., Wang J., Yao C., Zhang Z. (2019). Role of NKG2D and its ligands in cancer immunotherapy. *American Journal of Cancer Research*.

[25] Maude S. L., Frey N., Shaw P. A. (2014). Chimeric antigen receptor T cells for sustained remissions in leukemia. *The New England Journal of Medicine*.

[26] Giavridis T., van der Stegen S. J. C., Eyquem J., Hamieh M., Piersigilli A., Sadelain M. (2018). CAR T cell-induced cytokine release syndrome is mediated by macrophages and abated by IL-1 blockade. *Nature Medicine*.

[27] Tonn T., Schwabe D., Klingemann H. G. (2013). Treatment of patients with advanced cancer with the natural killer cell line NK-92. *Cytotherapy*.

